# Do employees benefit from collaborations between out of hours general practitioners and emergency departments?

**DOI:** 10.1186/s12913-018-2919-y

**Published:** 2018-02-17

**Authors:** Elisabeth Sybilla Johanna van Gils-van Rooij, Sjoerd Michael Broekman, Dingenus Herman de Bakker, Berthold Rudy Meijboom, Christoffel Joris Yzermans

**Affiliations:** 1 Scientific Centre for Care and Welfare (Tranzo), Tilburg University, Tilburg, The Netherlands; 2 GP co-operative Zuidoost Brabant, Eindhoven, The Netherlands; 3 The Netherlands Institute for Health Services Research (NIVEL), Utrecht, The Netherlands

## Abstract

**Background:**

In an attempt to redirect patients who are inappropriately attending hospital emergency departments (ED) and in doing so provide the right care at the right place, out-of-hours GP (General Practitioner) services and EDs increasingly collaborate in Urgent Care Collaborations (UCCs). Work satisfaction is an important factor in analysing the impact of this organisational change. The objective of this study is, firstly, to discover if there is a difference in the employee experiences between those working in UCCs and those in traditional out-of-hours services in which EDs and out-of-hours GP services operate separately (i.e. “usual care”). Secondly, we would like to identify which factors affect employees’ experiences in these settings.

**Methods:**

This study followed a cross-sectional study design, comparing usual care with UCCs. Data regarding employee experiences were collected from physicians, nurses, nurse practitioners, medical assistants and front desk personnel, by means of a questionnaire with scales regarding *quality, workload* and *co-operation between the out-of-hours GP service and ED*. Independent samples t-tests were used to determine mean differences between the settings. Multiple linear regression analyses were performed to test which items affected the perceived *quality, workload* and *co-operation*.

**Results:**

The results showed that mutual *co-operation* alone was perceived as significantly better in UCCs compared to usual care. If divided between employers, no differences were found in the employee experiences working in out-of-hours GP services. ED employees in UCCs experienced a significantly better *co-operation* with their GP colleagues than their peers in the usual care setting, but also a higher *workload*. Remarkably, ED employees were less satisfied in general. The multiple regression model showed that perceived *quality, workload* and *co-operation* were interrelated. *Co-operation* was the only aspect that was rated higher in the UCC setting.

**Conclusion:**

While perceived *quality* is equal and *co-operation between out-of-hours GP service and ED* is better, the objective and perceived ED *workload* was higher in UCCs compared to usual care. Though UCCs relieve the pressure on EDs concerning the number of patients, they seem to aggravate the *workload*. EDs need to be careful not to excessively adjust staff capacity when responding to lower numbers of patients.

## Background

Out-of-hours emergency care is generally regarded as one of the most onerous aspects of doctors’ and nurses’ work. Crowded waiting rooms, threatening, or even violent, situations as well as patients requesting for help although they do not require urgent care or may be treated by a General Practitioner (GP) [1–4], plus the inherent irregular hours which characterises this work, puts a heavy burden on health care workers in these settings. In this environment, work dissatisfaction may all too easily lead to a reduced quality of care and eventually burn out.

However, a new step has been taken recently in the organisation of emergency care in the Netherlands (Table 1: a brief overview of emergency care in the Netherlands) Intensified collaboration between out-of-hours GP services and emergency departments has been realised in so-called Urgent Care Collaborations (UCC). The primary goal of this organisational change is to promote the more efficient use of services, reducing the number of self-referred out-of-hours ED patients that present, in many cases, minor, non-urgent problems that generally can be treated by a GP or do not require treatment at all [4–7]. UCCs seem to succeed at this objective [8].

**Table 1:** Emergency care in the Netherlands [23–25]

Emergency care in the Netherlands is mainly provided by emergency departments (ED) and general practitioners (GP). During out-of-hours care, GPs mostly collaborate in out-of-hours services: large on-call rotations in which they take care of each other’s patients. In order to have access to hospital care, including EDs, patients are obliged to have a referral from an ambulant emergency service or GP, who functions as a gatekeeper. However, in practice, many patients attend the ED directly. Out-of-hours GP services and ED contacts are covered by obligatory health insurance. For hospital services (i.e. ED visits), there is a compulsory fee of at least €170 (at the moment of data collection). Prices of care pathways are determined by the national DTC system (DTC means the registered diagnosis and treatment combination). Out-of-hours GP services operate with one fixed budget, based on the catchment population, which is converted to a price per medical service (advice, consultation at care center, consultation at home). Since the 2006 Health Insurance Act, the Dutch healthcare system is based on a market of regulated competition. The prices for medical services are determined after negotiations between health insurance companies and care providers. As all citizens of the Netherlands are required to have health insurance coverage, every citizen pays for annual healthcare expenditures.	

This organisational change may challenge work satisfaction. The introduction of out-of-hours GP services during the 2000s in the Netherlands was associated with higher levels of work satisfaction among GPs compared to the rota system [9]. Therefore we ask: How do health care professionals now regard working in UCCs?

### Urgent care collaborations

While patients in usual care settings decide for themselves to contact an out-of-hours GP service or an ED, UCCs offer an intensified collaboration between out-of-hours GP services and EDs. They remain separate organizations with different registration systems but work closely together. They have one, combined entrance, front office desk and telephone number. Patients are allocated to either the out-of-hours GP service or ED based on a system of triage. In UCCs triage is performed by a medical assistant (a health care professional that supports the work of a GP by performing routine tasks and procedures, triage and patient scheduling) or nurse, using the Netherlands Triage System (NTS) [10]. Allocation to the out-of-hours GP service can result in medical advice, if possible by telephone, or a consultation with a GP at the care centre or patient’s home. If necessary, the GP can still refer a patient to the ED. Currently, UCCs operate solely out-of-hours.

In the usual care setting the out-of-hours GP services and EDs each use their own system of triage. At the out-of-hours GP services participating in this study, triage is performed by a medical assistant (a health care professional that supports the work of a GP by performing routine tasks and procedures, triage and patient scheduling), using the Netherlands Triage System (NTS) [10] or Telephone Advice System (TAS). Within the EDs triage is performed by a nurse, using the Manchester Triage System (MTS) or Emergency Severity Index (ESI). MTS and ESI are the most frequently implemented five-level triage systems in The Netherlands [11] . Out of hours GP services and UCCs only operate during out of hours. During office hours, patients attend their own GP. In order to have access to hospital care, including EDs, patients are obliged to have a referral from a GP or ambulant emergency service. However, in practice patients can attend the ED directly.

There is a growing tendency towards UCCs [12–14], because it is considered to be a chance to redirect inappropriate ED attenders and provide the right care at the right place. Evaluations and studies indicate that in UCCs fewer patients attend the ED, while out-of-hours GP services handle more patients [8, 14, 15]. This is reflected in the patient population. In UCCs relatively more very urgent and/or complicated health problems are treated by the EDs than in usual care settings [8, 16].

### Employees’ experience

UCCs intend to provide patients with the most suitable treatment in order to improve the efficiency of emergency care with at least the same quality as perceived in usual care, and improve co-operation between the out-of-hours GP service and ED. Given these intentions and the possible effect on workload, it is important to analyse the impact of the organisational change with respect to these factors.

The perceived *quality of care* may be affected by the implementation of UCCs. Employees observe the patient population and may value, more, the quality of care in UCCs, as patients are pre-sorted more accurately; the perceived *co-operation between out-of-hours GP services and EDs* might be higher in UCCs due to the more explicit collaboration and the architectural design of UCCs.

Changes in the patient population can affect the perceived *workload*. A higher workload can be expected for GPs and medical assistants (a health care professional who supports the work of a GP by performing routine tasks and procedures, triage and patient scheduling). This is because they have to deal with more patients while their capacity is not adjusted sufficiently to the new situation. The effect regarding workload for ED employees is not straightforward. On the one hand workload may be lower because the number of patients decreases, while on the other hand they may experience a higher workload because they have to deal with relatively more very urgent and/or complicated health problems. In addition, if EDs are cutting staff as they prepare for lower numbers of patients, this can influence the perceived workload.

Moreover, we expect that the perceived *quality of care*, *workload* and *co-operation between out-of-hours GP services and EDs* are interrelated. *Quality of care* may be valued higher in the UCC setting due to more *co-operation between out-of-hours GP services and EDs*. Increased or decreased *workload* might jeopardise the perceived *quality of care*, as stress and stress-related illnesses such as burnout seem to be associated with work performance, lower patient satisfaction and longer patient-reported recovery time [17–19]. At the same time, professionals might accept a higher objective *workload* when they feel it improves the *quality of care* and *co-operation between out-of-hours GP services and EDs*.

Among the available literature, we found several studies evaluating some level of employee experience in UCCs during out-of-hours shifts. However, these previous studies did not study UCCs as a whole, nor focus on the perceived *quality of care* and perceived *co-operation between out-of-hours GP services and EDs*. Kool et al. [14] found that employees working in UCCs were less satisfied than their colleagues working in out-of-hours GP services or EDs. This conclusion was drawn in relation to the following human resource topics: autonomy, social climate, information provided by the organisation, the culture of the organisation, satisfaction with their work and the possibility of using their own capacities. In a study among GPs, Van Uden et al. [20] identified that GPs from the separated model were generally more satisfied with the organisation of out-of-hours care than GPs from the collaboration model. However, the co-operation experienced with medical specialists was much better in the UCC setting than in the usual care setting. A study by Sturms et al. [15] that measured changes in the workload experienced after implementation of an UCC in a Dutch hospital, showed an overall increase in workload for GPs and their medical assistants and a decreased workload for ED employees during nights. However, changes in workload did not lead to significant differences in the level of satisfaction regarding these workloads.

### Research questions

As UCCs intend to provide patients with the most suitable treatment in order to improve the efficiency of emergency care with at least the same quality as perceived in usual care, and improve co-operation between the out-of-hours GP service and ED, while changes in patient flows possibly affect workload, employees’ experience is an important outcome when evaluating this organisational change. However, it remains unclear to what extent perceived *workload*, *quality of care* and *co-operation between out-of-hours GP services and EDs* differ in a UCCs compared to usual care. This study aims to identify these differences and explore the relationship between *workload*, *quality of care*, *co-operation between out-of-hours GP services and EDs* and employer and employee characteristics. This is important because the effects may differ between employers, that is the out-of-hours GP services or EDs and the employees, in particular with regard to their profession, age, and gender.

We address these two research questions: 1. Are, the perceived *quality of care, workload* and *co-operation between out-of-hours GP services and EDs* different in UCCs, compared to usual care? 2. Which factors affect the perceived *quality, workload* and *co-operation between out-of-hours GP services and EDs?*

## Methods

This study followed a cross-sectional study design, comparing usual care with UCCs. In the usual care setting out-of-hours GP services and EDs worked separately. The usual care setting consisted of three regions, with an adherent population of 538,000 residents and in total 751 employees. This UCC setting comprised three regions in which UCCs have been adopted, 533,000 residents and, in total, 577 employees.

All the regions participating are rural as well as urban locations situated in the south-eastern part of the Netherlands.

### Study population

The study population consisted of employees of both the EDs participating and of the out-of-hours GP services. The UCCs in this study were launched between December 2008 and March 2009. Physicians, nurses, medical assistants and front desk personnel were included in this study. The physicians comprised GPs, residents (junior doctors in medical training) and medical specialists (hospital consultants) in the specialisms general surgery, cardiology, internal medicine and orthopaedics. We chose to invite only the medical specialists and residents from these specialisms because these are the professions most consulted within EDs.

### Data

#### Questionnaire

Data regarding employee experiences were collected from all employees by means of a questionnaire. We chose to develop a questionnaire based on a validated questionnaire for GPs and medical assistants working within out-of-hours GP services [21] and a work satisfaction questionnaire designed for nurses [22]. The questionnaire contained four sets of topics: (a) *overall work experience*, (b) *workload*, (c) *quality of care* and (d) *co-operation between out-of-hours GP services and EDs*. Four-point Likert scales were used for two items (‘employee involvement’ and ‘recommendation of organisation to acquaintance’). Five-point Likert scales were used for all other items. The use of both four- and five-point Likert originates from the original questionnaires used as input for this questionnaire. The survey was available online and could only be accessed using a personal link sent by email. Filling in the questionnaire took, at the most, 10 min. Employees (total: *n* = 1309, usual care: *n* = 752, UCCs: *n* = 557) were invited to participate in this study by their employer through a standard email message including the personal link that was drawn up by the study researchers. Non-responders received a reminder within 7 days. Data were transcribed to SPSS automatically from the online survey. Item scores of reverse-scored questions were recoded.

The questionnaire was subjected to a factor analysis (Table 2). A scree test was used to identify the number of the questionnaire’s underlying factors. This revealed a first point of inflexion after the third component, three factors contributed the most to explaining the variance in the dataset. Therefore, it was chosen to retain three factors for further investigation. The three factor component solution explained a total of 38.6% of the variance.

**Table 2:** Factor analysis, structure matrix

	*Quality of care*	*Workload*	*Co-operation*
Employee involvement	0.403		
Pleasant organisation	0.474	0.376	
Quality of work front desk personnel	0.546		
Quality of work residents ED	0.550		−0.352
Quality of work medical specialists ED	0.480		
Quality of work nurses ED	0.363		
Quality of work assistant out-of-hours GP service	0.690		
Quality of work nurse out-of-hours GP service	0.651		
Quality of work ambulance driver out-of-hours GP service	0.469		
Quality of work GP	0.531		
Quality of the organisation	0.651		
Quality of physical triage	0.575		
Quality of triage by telephone	0.587		
Quality of patient care	0.644		
Quality, patient safety	0.508		
Quality of care inadequate	0.499		
Recommendation of organisation to acquaintance	0.446		
Workload during work at the care centre		0.799	
Workload during home visits		0.513	
Workload during work at the call centre		0.512	
Workload due to the amount of contacts		0.798	
Workload due to self-referrals		0.545	
Workload due to less urgent or unnecessary patients		0.425	
Workload due to very urgent or complex patients		0.326	
Workload due to triage		0.404	
Workload due to arranging hospital admission		0.315	
Workload due to arranging admission outside the hospital		0.333	
Sufficient time for accurate patient care	0.354	0.581	
Sufficient time to discuss patient care problems with colleagues		0.540	
Sufficient time for direct patient care	0.338	0.520	
Quality of work and available time		0.684	
Co-operation out-of-hours GP service – ED, differences in culture/perspective	0.367		−0.593
Co-operation out-of-hours GP service – ED, quality of medical performance	0.503		−0.564
Co-operation out-of-hours GP service – ED, referral	0.392		−0.549
Co-operation out-of-hours GP service – ED, feedback after referral			−0.601
Co-operation out-of-hours GP service – ED, meeting			−0.788
Co-operation out-of-hours GP service – ED, co-operation colleagues			−0.843
Co-operation out-of-hours GP service – ED, acquaintance with colleagues		0.324	−0.559
Co-operation out-of-hours GP service – ED, patient flow	0.382		−0.553
Co-operation out-of-hours GP service – ED, efficiency	0.453		−0.552
Co-operation out-of-hours GP service – ED, right care at right place	0.408		−0.511
Variance explained (%)	22.115	9.422	7.026
Eigenvalues	9.067	3.863	2.881
Total number of items	17	14	10
Alpha	0.90	0.84	0.87

Factor loadings less than 0.300 are suppressed. Bold text: main factor loading

We used the Direct Oblimin method for oblique rotation in order to find the proper factor solution within the data as we expected factors regarding the four topics of the survey to correlate. A loading of an absolute value of more than 0.3 was considered to be important. The questionnaire showed high loadings on one factor and small loadings on other factors. Our interpretation lead to three scales: *quality (of care delivered), workload (due to the amount of contacts and their complexity)* and *co-operation between out-of-hours GP services and EDs (regarding patient flow effectiveness and individual contact between professionals)*. The scales *quality* and *workload* showed a correlation of 0.28; *quality* and *co-operation* 0.38; *workload* and *co-operation* 0.26. Reliability analysis indicated that removing items from the scales would not improve the overall reliability of the scale. All scales showed Cronbach’s alpha values exceeding 0.80.

Employees who answered fewer than five questions from the survey were considered non-respondent. Scale scores were computed for each case, provided that 50% of the answers within one factor were present. By summarising the items scores and dividing this by the number of items, scale scores between 0 and 5 were calculated. Higher scores correspond with more perceived *quality,* higher *workload* and more *co-operation between out-of-hours GP services and EDs.*

#### Employee and employer characteristics

Gender, age and profession were also assessed by means of the questionnaire. Profession was reduced to two clusters: physicians and support staff. The cluster physicians comprised GPs, residents and medical specialists. Nurses, nurse practitioners, medical assistants and front desk personnel complete the cluster support staff.

The management of the out-of-hours GP services and EDs forwarded the number of physicians and support staff deployed per shift (staffing level); the number of contacts per shift was obtained from routinely kept medical records. This information was combined to calculate the number of contacts per employee per shift.

### Analysis

Before addressing the research questions, information regarding the response on the questionnaire, employee characteristics and staffing were analysed using descriptive statistics. To test for differences between settings, chi-square and independent samples t-tests were used.

In order to answer research question one (Are, the perceived *quality of care, workload* and *co-operation between out-of-hours GP services and EDs* different in UCCs, compared to usual care?) independent samples t-tests were, after testing for normality, used to determine mean differences in the experienced *quality, workload* and *co-operation* in of out-of-hours GP services and ED employees between settings.

To test which aspects affected perceived *quality*, *workload* and *co-operation between out-of-hours GP services and EDs* (research question two), three separate multiple linear regression analyses were performed.

A p-level of less than 0.05 was considered to be statistically significant. All data were analysed using SPSS statistics, version 20.

## Results

In total 1309 employees were invited to fill in the online questionnaire, of whom 752 were from the usual care setting and 557 the UCC setting (Table 3). In the usual care setting 341 (45%) employees responded; 240 (43%) in the UCC setting. In both settings the response was higher among support staff members compared to physicians (60% vs. 35%). Significantly more (65% vs. 52%) support staff members responded in the usual care setting than in UCCs.

**Table 3:** Response in usual care vs UCC-setting

	Total		Usual care		UCCs	
	*n*	responders (%)	*n*	responders (%)	*n*	responders (%)
Total	1307	581 (44.4%)	751	341 (45.3%)	557	240 (43.1%)
Physician*	797	276 (34.6%)	459	150 (32.7%)	338	126 (37.3%)
Support staff	510	304 (59.6%)	292	191 (65.4%)	218	113 (51.8%)

Data are *n* followed by response within group. *statistically significant (*p* < 0.05) difference between usual care and UCCs

Table 4 shows the employee characteristics in UCCs and usual care. Overall, the proportion of physicians was significantly larger in UCCs compared to the usual care setting. Also, the percentage of male staff in the EDs was higher in the UCC than in the usual care setting. No significant differences were found in out-of-hours GP services staff between both settings.

**Table 4:** Employee characteristics for employees who returned the questionnaire in usual care and UCCs

	Total		Out-of-hours GP services		EDs	
	Usual care	UCCs	Usual care (*n* = 216)	UCCs (*n* = 141)	Usual care (*n* = 125)	UCCs (*n* = 99)
Profession*‡						
Physician	150 (44.0%)	126 (52.7%)	128 (59.3%)	92 (65.2%)	22 (17.6%)	34 (34.7%)
Support staff	191 (56.0%)	113 (47.3%)	88 (40.7%)	49 (34.8%)	103 (82.4%)	64 (65.3%)
Gender‡						
Male	115 (33.7%)	93 (38.8%)	94 (43.5%)	56 (39.7%)	21 (16.8%)	37 (37.4%)
Female	226 (66.3%	147 (61.2%	122 (56.5%)	85 (60.3%)	104 (83.2%)	62 (62.6%)
Age (mean ± SD)	43.79 ± 11.88	43.59 ± 11.10	45.4 ± 11.7	44.9 ± 11.2	41.04 ± 11.8	41.7 ± 10.7

*statistically significant (*p* < 0.05) difference between usual care for total population; †statistically significant (*p* < 0.05); ‡statistically significant (*p* < 0.05) difference between usual care and UCCs for ED employees

To assess the impact on *workload* it is important to analyse the differences in staff capacity between the settings. Therefore, information regarding the number of physicians, nurses, nurse practitioners, medical assistants and front desk personnel per shift were requested from the study locations and combined with the number of contacts per shift (Table 5). There were no differences between the settings for both GPs and support staff members of both ED and out-of-hours GP services. However, there is a substantial difference between usual care and UCCS, regarding the average number of contacts per ED physician. Overall, ED physicians at UCCs had to deal with more contacts per shift than their colleagues in the usual care setting (on average 12 vs. 7). More detailed analysis (not in table) showed that this overall pattern was consistent among the locations involved.

**Table 5:** Objective workload clustered by setting

	Out-of-hours GP services		EDs	
	Usual care	UCC	Usual care	UCC
All employees	12	12	3	3
Physicians	19	20	7	12
Support staff member	33	32	4	4

Data are number of patients per employee per shift

### Perceived quality, workload and co-operation

Table 6 shows the overall results of the scales, *quality, workload* and *co-operation between GPs and EDs,* for employees of the out-of-hours GP services and EDs in both settings. When we look at total staff (out-of-hours GP services and ED together) the perceived *co-operation between out-of-hours GP services and EDs* was overall rated higher in the UCC setting than in usual care. The other scales showed no significant differences between settings.

**Table 6:** Perceived quality, workload and co-operation, mean scores split by setting and care provider

	Item	Usual care		UCCs		Mean difference (95% CI)
		*n*	mean ± SD	*N*	mean ± SD	
Total	Quality	313	3.55 ± 0.42	226	3.54 ± 0.43	−0.013 (− 0.084 to 0.058)
	Workload	329	2.83 ± ± 0.53	222	2.92 ± 0.62	0.084 (−0.013 to 0.181)
	Co-operation	280	3.21 ± 0.52	216	3.38 ± 0.56	0.177 (0.080 to 0.273)
Out-of-hours GP services	Quality	206	3,57 ± 0,42	133	3,58 ± 0,42	0.012 (− 0.153 to 0.074)
	Workload	206	2,77 ± 0,56	132	2,65 ± 0,53	−0.120 (− 0.239 to 0.003)
	Co-operation	171	3,27 ± 0,55	123	3,39 ± 0,58	0.122 (−0.009 to 0.254)
ED	Quality	107	3,52 ± 0,38	93	3,48 ± 0,43	−0.040 (− 0.153 to 0.074)
	Workload	123	2,94 ± 0,47	90	3,31 ± 0,52	0.367 (0.233 to 0.501)
	Co-operation	109	3,11 ± 0,46	93	3,37 ± 0,52	0.264 (0.129 to 0.400)

Scored 1–5, higher scores correspond with more perceived *quality* (1 = bad, 5 = excellent), higher *workload* (1 = workload not considered a burden, 5 = high burden) and more *co-operation between GPs and EDs* (1 = very dissatisfied, 5 = very satisfied)

When separated by employer – EDs and out-of-hours GP services – it stands out that no differences were found for out-of-hours GP services employees. However, ED employees experienced a significantly better *co-operation* with their GP colleagues than their peers in the usual care setting, but also a higher *workload*.

### Factors associated with quality, workload and co-operation

Table 7 shows the results of a multiple regression analysis in which the different scales are related to each other and to casemix variables. *Co-operation between out-of-hours GP services and EDs* is significantly related to setting, when controlled for casemix variables. So, employees in UCCs experienced significantly better co-operation when corrected for all other variables (Table 7).

**Table 7:** Multiple linear regression analyses of perceived quality, co-operation and workload by setting casemix-characteristics and other employee experience factors

		Quality	Workload	Co-operation between out-of-hours GP services and EDs
Beta coefficients	Setting^a^	−0.059	0.052	0.140*
	Employer^b^	−0.156*	0.303**	−0.137*
	Gender^c^	0.003	0.068	−0.002
	Age	−0.084	0.026	−0.017
	Profession^d^	0.062	−0.247**	−0.029
	Quality	–	0.272**	0.360**
	Co-operation	0.299**	0.188**	–
	Workload	0.352**	–	0.212**

^a^0 = usual care, 1 = UCCs; ^b^0 = out-of-hours GP services, 1 = ED; ^c^0 = female, 1 = male; ^d^0 = support staff, 1 = physician; *Variable contributes significantly (*p* < 0.05) to the model; **Variable contributes significantly (*p* < 0.01) to the model

Higher scores correspond with more perceived *quality,* higher *workload* and more *co-operation between out-of-hours GP services and EDs*

The association between *quality*, *co-operation between out-of-hours GP services and EDs* and *workload* is apparent in the regression models. The perceived *quality, workload* and *co-operation* are significantly positively interrelated. Perceived better *quality* was associated with a higher perceived *workload* and better *co-operation*; *co-operation* is associated with higher perceived *quality* and *workload*; higher workload is associated with better perceived *co-operation* and *quality*.

If we look at all scales then employees in EDs were less satisfied, the beta coefficients indicate that they perceived less *quality*, a higher *workload* and less *co-operation* than their colleagues working at the out-of-hours GP services. *Profession* only seems to influence *workload as* physicians perceive a lower *workload* compared to support staff.

## Discussion

### Are the perceived *quality of care*, *workload* and *co-operation* different in UCCs, compared to usual care?

Primarily, the aim of this study was to assess the impact on employee experience of UCCs compared to usual care in which EDs and out-of-hours GP services work separately. The overall results showed that it was only the perceived *co-operation between out-of-hours GP services and EDs* which was significantly better in UCCs compared to usual care when controlled for casemix variables.

Looking specifically at staff working in out-of-hours GP services, the results of this study showed no statistically significant differences. This is in contrast to the results of Van Uden et al. [20] and Sturms et al. [15] Van Uden et al. [20] revealed that *co-operation with medical specialists* was much more appreciated by GPs in the UCC setting as opposed to the usual care setting. A possible explanation for the difference in these results is that GPs, in both settings, were used to referring patients to the ED and were acquainted with the work of the ED (most out-of-hours GP services were located relatively close to each other – within five kilometers). The increased *workload* after the implementation of an UCC as revealed by Sturms et al. [15] is what ought to be expected, based on the assumption that staff capacity was not adjusted. However, in our study population both perceived *workload* and the average number of contacts per staff member, per shift were not different for out-of-hours GP services in UCCs compared to usual care. This implies that out-of-hours GP services staffing was tuned to the needs of the patient population and that they adjusted adequately to the organisational change.

ED employees in UCCs experienced a significantly better *co-operation with the out-of-hours GP service* and higher *workload*, compared to the usual care setting. A higher workload was not expected as patients were triaged, that is assigned appropriate care in advance, more accurately in UCCs. In this way patients were treated more often by an out-of-hours GP service and EDs did not have to deal with inappropriate self- referrals. Nevertheless, the perceived higher *workload* does correspond with differences in patient characteristics. For example, the EDs have to deal with more very urgent and/or complicated health problems in UCCs [8], which may account for a higher perceived *workload*. Moreover, the average number of contacts per ED physician per shift was – as well as the *workload* experienced – considerably higher in UCCs. This suggests that ED staffing may not be adequate in the UCC setting. Possibly the reduction in ED staffing in order to anticipate to the changing patient population, was too severe. The intensified collaboration in UCCs seemed to have a positive influence on the *co-operation between out-of-hours GP services and EDs* which was experienced, as the *co-operation* outcome was significantly higher in this setting.

### Which factors affect perceived *quality, workload* and *co-operation?*

Multiple regression analyses showed that *co-operation* as well as *workload* were positively associated with *quality*. It seems remarkable that *workload* was positively associated with *quality*. However, the review by Muse et al. [19] showed that several psychological studies support theories that exceedingly low and high levels of stress decrease job performance. Possibly, the range of perceived *workload* in this population was not so high that it leads to excessive stress and lower *quality.* Also, *co-operation between out-of-hours GP services and EDs* and *workload* were linked: professionals that perceive a high *workload* also perceive more *co-operation*. However, any causal inferences cannot be drawn from this association. Providing good quality of care and good collaboration require time, which could lead to a higher perceived workload.

The employer (out-of-hours GP services or ED) also appeared to affect the employee experience outcome. It seems that ED employees were less satisfied in general. They perceive less *quality* and *co-operation* plus a higher *workload* than employees of the out-of-hours GP services. This is a remarkable outcome because the collaboration between out-of-hours GP services and EDs was meant to relieve pressure on EDs. Support staff members in particular experienced a significantly higher *workload* than physicians. They seem to be confronted with a situation in which fewer physicians were available, while the cases were more complex.

*Co-operation* was the only scale influenced by setting when corrected for casemix variables (employer, gender, age, profession and the other scales). *Co-operation* is perceived as significantly better in UCCs than in usual care.

### Strengths, limitations and implications for further research and clinical practice

To our knowledge this is the first study concerning the employee experience in UCCs to focus on the factors *quality*, *workload* and *co-operation* from the perspective of staff working in EDs and out-of-hours GP services taking into account both perspectives. We consider it a strength of the study that the study population reflects the workplace and that input is collected from all levels of employees working within the GP service and ED. This offers a broad perspective on the experienced quality of care, workload and co-operation between the ED and out-of-hours GP service. The choice to use one questionnaire for all staff contributes to this, as it makes it possible to look at the results as a whole and to make a distinction between organisations (out-of-hours GP service/ED) and profession (physicians/support staff). In addition, completing the survey was simple and fast as a result of which the response was reasonably high.

Moreover, three UCCs and three usual care regions participated, which enhances the degree to which they are generally applicable. It should be noted that the UCCs studied were among the early adopters of this innovation. Many other regions followed, choosing varying models of co-operation. All different approaches have in common that the co-operation between the out-of-hours GP service and ED is enforced and that patients are redirected to the GP service. A weakness of this study is that data collection should, preferably, have taken place before and after the implementation of an UCC.

We suggest performing more research with the questionnaire we have developed, (a) in order to examine whether differences in *perceived workload* remain if *objective workload* is equal, and (b) to compare UCCs. Furthermore we see a higher *objective workload* among physicians in emergency departments in UCCs and a lower satisfaction among support staff. This suggests an over adjustment to the situation, in which a larger part of the patient flow is directed to out-of-hours GP services leaving the ED with more complex cases and fewer physicians available. Whether this is indeed how it works requires further study. Meanwhile, *workload* has to be monitored closely, as the results indicated a rather high *perceived workload* whereas previous studies demonstrate that the quality of care as well as employee well-being can be affected by high levels of stress.

## Conclusion

UCCs were established to promote the more efficient use of services, thereby reducing the number of inappropriate self-referred out-of-hours ED patients. They seem to have succeeded in this [8]. However, the results regarding work satisfaction are ambiguous. While the perceived *quality* is equal and *co-operation between out-of-hours GP services and ED* is better – which was a secondary target of UCCs – the objective and perceived ED *workload* was higher in UCCs compared to usual care. While UCCs relieve pressure on EDs concerning the number of patients, they seem to aggravate *workload*. EDs need to be careful not to overadjust staff capacity when responding to lower numbers of patients.

## Abbreviations

ED: Emergency department
ESI: Emergency Severity Index
GP: General Practitioner
MTS: Manchester Triage System
NTS: Netherlands Triage System
TAS: Telephone Advice System
UCC: Urgent Care Collaboration
